# Book Reviews

**Published:** 1816-11

**Authors:** 


### CRITICAL ANALYSIS
### *OF RECENT PUBLICATIONS*
IN THE
### DIFFERENT BRANCHES OF PHYSIC, SURGERY, AND MEDICAL PHILOSOPHY.

*Essays on Hypochondriacal and other Nervous Affections.* By John Reid, M.D. Member of the Royal College of Physicians, London; and late Physician to the Finsbury Dispensary. 8vo. pp. 272. Longman and Co. 1816.

THE abilities of Dr. Reid are too well known to require any encomium from us. His labours in the Monthly Magazine attracted very general attention, by the popular form in which they appeared, as well as from their intrinsic merit. We could have been glad to have seen them all collected in the manner that Dr. Willan's were, after he relinquished the office of reporter for that valuable miscellany; but, but, on this subject, we shall transcribe the author's advertisement.

"It is right (says he) to apprize the reader of the following Essays, that many passages of them have been taken, without much alteration, from the Medical Reports, which, after Dr. Willan had relinquished the task, I was, for a course of years, in the practice of communicating to the Old Monthly Magazine.

"It was my original design to have endeavoured to write something more systematic and complete on the subject of mental diseases; but domestic circumstances, in which the public are not interested, having interfered with the prosecution of that object, I have been induced to commit to the press, in the form of Essays, what I had regarded as materials merely towards the formation of a larger and more methodical work."

We sincerely hope that as the cause to which Dr. Reid alludes may gradually lessen in its effects, the public will receive the accomplishment of the larger work. Of the present, from the disjointed form of Essays, in which it appears, it is difficult to give a succinct account. We must, therefore, content ourselves with a few selected extracts, and a general table of the whole. By these the reader will be able to judge of the execution, and learn the contents.

The first essay, "on the influence of the mind on the body," should be constantly kept in view, especially by practitioners in the metropolis and in other large towns. In more sequestered spots, not only the history, but the moral as well as physical character, and, most of all, the external circumstances of the patient, are pretty generally known. But, in a larger circle, the patient is often a stranger to the physician, and, perhaps, conceals the most important circumstances in the history of his case—more particularly those corroding, those cankering distresses of the mind, which, in the late unsettled state of the mercantile world, have been the source of so many diseases. All this is extremely well expressed in a chapter, which concludes with some judicious remarks on the probably greater equality of happiness among mankind than the variety of rank would seem to indicate.

The next essay, "on the power of volition," contains sentiments so important, so often overlooked, and so well expressed, that we shall select a part of it as a specimen of the manner in which the whole is conducted. It also contains a history which we are glad of this opportunity of recording.

"We often act on the ill-founded idea that nervous complaints are altogether dependant upon the power of the will; a notion which, in paradoxical extravagance, scarcely yields to the doctrine of a modern, though now obsolete writer, on the Philosophy of Morals, who asserted that no one need die, if, with a sufficient energy, he *determined* to live. To command, or to advise a person labouring under nervous depression, to be cheerful and alert, is no less idle and absurd, than it would be to command or advise a person, under the direct and most intense influence of the sun's rays, to shiver with cold; or one who is 'wallowing naked in December's snows,' to perspire from a sensation of excessive heat. The practice of laughing at or scolding a patient of this class, is equally cruel and ineffectual. No one was ever laughed or scolded out of hypochondriasis. It is scarcely likely that we should elevate a person's spirits by insulting his understanding. The malady of the nerves is, in general, of too obstinate a nature to yield to a sarcasm or a sneer. It would scarcely be more preposterous to think of dissipating a dropsy of the chest, than a distemper of the mind, by the force of ridicule or rebuke. The hypochondriac may feel, indeed, the edge of satire as keenly as he would that of a sword; but, although its point should penetrate his bosom, it would not be likely to let out from it any portion of that noxious matter by which it is so painfully oppressed. The external expression of his disorder may be checked by the coercive influence of shame or fear; but, in doing this, a similar kind of risque is incurred as arises from the repelling of a cutaneous eruption, which, although it conceal the outward appearance, seldom fails still more firmly to establish the internal strength, to increase the danger, and to protract the continuance of the disease. By indirect and imperceptible means, the attention may, in many instances, be gently and insensibly enticed, but seldom can we with safety attempt to *force* it from any habitual topic of painful contemplation. In endeavouring to tear the mind from a subject to which it has long and closely attached itself, we are almost sure to occasion an irreparable laceration of its structure.

"However well founded may be these observations, it must still be acknowledged that the different degrees of power which persons of various habits and constitutions appear to possess, not only over the feelings and faculties of the mind, but likewise over what are called the involuntary muscles, and even the blood vessels of the body, may afford ground for an inquiry, curious at least, if not important, how far so desirable a power may be acquired, and to what extent, by some yet undiscovered method of education, it may be elevated and improved.

"Dr. Cheyne, in one of his medical treatises, narrates a case, the accuracy of which is established by an irrefragable combination of evidence, of a man who could die to all appearance, at any time that he chose, and, after having lain for a considerable time exactly as a corpse, was able, as it should seem, by a voluntary struggle, to restore to himself the appearance and all the various functions of animation and intellect. It is to be inferred from the latter part of the story, that the unnatural and painful exertions by by which this person assumed the semblance of decease, produced at length a fatal result. Death would be no longer mocked with impunity. The counterfeit corpse, a few hours after its last revival, relapsed into a state which was capable of no subsequent resuscitation. But the case is so interesting and remarkable, as to deserve our giving it in all the detail with which Dr. Cheyne presents it to his readers.

" 'He could die or expire when he pleased, and yet by an effort, or somehow, he could come to life again. He insisted so much upon our seeing the trial made, that we were at last forced to comply. We all *three* felt his pulse first; it was distinct, though small and thready, and his heart had its usual beating. He composed himself on his back, and lay in a still posture for some time; while I held his right hand, Dr. Baynard laid his hand on his heart, and Mr. Skrine held a clear looking-glass to his mouth. I found his pulse sink gradually, till at last I could not feel any by the most exact and nice touch. Dr. Baynard could not feel the least motion in the heart, nor Mr. Skrine perceive the least sort of breath on the bright mirror he held to his mouth. Then each of us, by turns, examined his arm, heart, and breath, but could not, by the nicest scrutiny, discover the least symptom of life in him. We reasoned a long time about this odd appearance as well as we could, and finding he still continued in that condition, we began to conclude that he had indeed carried the experiment too far, and at last we were satisfied he was actually dead, and were just ready to leave him. This continued about half an hour. By nine o'clock in the morning in autumn, as we were going away, we observed some motion about the body, and upon examination found his pulse and the motion of his heart gradually returning: he began to breathe gently and speak softly. We were all astonished to the last degree at this unexpected change, and, after some further conversation with him and with ourselves, went away fully satisfied as to all the particulars of this fact, but not able to form any rational scheme how to account for it. He afterwards called for his attorney, added a codicil to his will, &c. and calmly and composedly died about five or six o'clock that evening.'*

" Burton, in his Anatomy of Melancholy, reports cases which were somewhat similar, but by no means equally wonderful with the preceding. 'Celsus speaks of a priest that could separate himself from his senses when he list, and lie like a dead man, void of life and sense. *Qui, quoties volebat, mortuo similis jacebat, auferens se a sensibus.* Cardan brags of himself, that he could do as much, and that when he list.'†

" Such instances serve to shew that the will can perform wonders in the controul and management of our corporeal frame. If such an extraordinary degree of command be possible, as has been her

" * Cheyne's English Malady.

" † Burton's Anatomy of Melancholy, vol. i. p. 134. 8vo. edit."

 represented, represented, it is fair to conclude that we may have, in general, a greater power than we are aware of over the animal and vital functions. If, by a determination of the mind, it be practicable in some cases to suspend altogether the appearance of life, it is reasonable to believe, that, by the same means, we may put at least a temporary stop to the symptoms of disease. We would not be paradoxical or extravagant enough to assert, that, for a person to be in health, it is sufficient that he wills it; but, without transgressing the moderation of truth, we may venture to give it as our opinion, that a man often indolently bends under the burden of indisposition, which a spirited effort would, in the first instance, have shaken from his shoulders. If, upon the approach of the malady, he had resolutely set his face against it, he would probably have arrested it in its threatened attack.

"I was once consulted concerning an hypochondriacal lady, who complained principally of an invincible indolence and languor. She seemed almost incapable of voluntary motion. This apparent incapacity had been sanctioned and confirmed by authority as well as indulgence. She had been told by a very complaisant physician that 'exertion would be poison to her,' and had too literally reposed under the shelter of that professional opinion. Many, from an anxiety to avoid this falsely imagined poison, reject the most effectual antidote to the real miseries of life, as well as to a large proportion of its diseases. To a patient, however, whose malady is lassitude, exertion should be prescribed at first only in very small doses. Such a person would be apt to be exhausted even by an ordinary task of exercise, and might thus be discouraged from further efforts at activity.

"In the class of what are called nervous affections, it unfortunately happens that the very essence of the disease often consists in a debility of the resolution; that the ailment of body arises from an impotency of spirit, a palsy of the power of resistance. A malady, occasioned by the weakness of the mind, is not likely to be cured by its energy. A tendency to sickness of the stomach may often be overcome by striving against it; but a squeamish disgust of life cannot, in the same degree, be counteracted by a similar kind of exertion. It is not uncommon to say to a drooping or desponding valetudinarian, 'only exert yourself, and you will get the better of your complaint;' whereas, in many instances of this kind, it might as well be said to an invalid confined to his bed by a paralysis of his limbs, only run or walk and you will be well. People, in general, are apt to think that a man, under the weight of constitutional or habitual melancholy, may keep up his spirits as a little miss can hold up her head, upon merely being *bid* to do so.

"It is often as impossible for an hypochondriac, by any voluntary effort, to get the better of his complaint, as for a man of ordinary stature to gain an ascendancy, when struggling under the compression of a giant."

The

The third essay, on "the fear of death," contains more novelty than we could have expected on such a subject, and even some directions for the relief of this form of melancholy, which, if not new, have not occurred to us before.

The fourth essay is "on pride," and, like most of the others, partly moral, though all directed to physical objects.

The fifth is "on remorse," which may be considered, for the most part, only one form of hypochondriasis, unfortunately affecting those principally who have the least reason to reproach themselves.

The succeeding one, "on solitude," shews, in a very pointed manner, the necessity of cultivating social and industrious habits.

The seventh essay, "on excessive study, or mental application," we deem peculiarly important, for, whatever may be thought of the frivolity of the age, it is certain that there is more close application than at any former period of our history. The extraordinary talents required at the bar, in the pulpit, and, most of all, in the senate, are a constant stimulus to every enterprizing youth, and, unfortunately, its effect is greatly increased by the ambition of families of every description above those consigned to mere mechanical employments. The consequence of this is often an inattention to the moral character, and still more to the health, of the rising generation. This chapter is enriched with the case of a studious youth, who, from the apprehension of losing some academic honours, the acquisition of which he had anticipated, became, for a time, nearly insane. He, however, recovered. We could relate a less fortunate instance, and one which has made us doubt the real value of academical prizes, unless confined to poetry. If one person is elated, how many are distressed; and we may ask further, is not the acquisition of an expected honour frequently the harbinger of future inattention, partly from the intensity of previous study, and partly from a supposed superiority, which requires no longer any application whatever. Meanwhile the less successful candidates are dissatisfied either with themselves or with those who have the distribution of the prizes; and sources of jealousy, and even hatred, invade a sanctuary which should be unalloyed by such dangerous intruders. The author's instructions at the conclusion of this chapter cannot be expected to be altogether new: they are, however, very judiciously selected. In justice to our English universities, we ought to remark, that the examinations are now more judiciously arranged, so that there is less danger from honours which are open to all; but the prizes are still often attended with ill consequences.

Essay

Essay VIII. is entitled—"Vicissitude a cause and characteristic symptom of intellectual malady." If there is any chapter to which we should object it is to this; first, which may seem odd, because it is too short, that is, we conceive it might have been incorporated with some other; but most because it abounds too much with allusions not strictly medical. By this time our readers must have perceived in our remarks on many occasions what, perhaps, some may call a prudery in whatever relates to language. We are ready to acknowledge that this fastidiousness ill accords with some of the figures we meet with in the notes, as well as in the text, at the beginning of this chapter. We are ready to impute this to a richness of imagery, which, if we judge rightly, the author is incapable at all times of concealing. It becomes us, at however, to acknowledge that it has its attendant advantages of constantly keeping the attention alive, by well-arranged periods and well-selected phrases; of which the concluding part of this very chapter furnishes a striking example. It may be added that this is the most important part, as it contains the mode of treatment.

The chapter on the "want of sleep" follows. On this occasion we shall offer our own remedy, in addition to bathing, which is proposed by our author; and the value of which we are ready to admit. Where the mind is so engaged on a single object as to prevent sleep, we have found a novel, not too interesting, but sufficient to disengage the patient from his former train of thoughts, a most certain remedy. It is not merely the interest he takes in the history, but the world of fiction into which he is now introduced, will often disengage him from the objects around him, or make him think less of them, from a momentary absence, or sometimes from the superior grandeur of the characters with which he now seems conversing. Nor ought we to forget the celebrated remedy, the pillow of hops, which first closed the watchful eyes of our unfortunate monarch!

The following chapter, on "intemperance," is full of good things, and the term very properly extended beyond the use of wine or ardent spirits. Opium and its effects meet with due consideration, which is the more important, as it is very generally admitted that recourse to this dangerous drug has increased in proportion as indulgence in wine has diminished among us. This chapter includes also the sudden acquisition of wealth among the stimuli, equally injurious with the former. The author asserts, with much truth, that there is little danger to the mind from a depression of external ternal circumstances, compared to what often arises from sudden elevation.

It is next shewn that the extreme abstinence to which some hypochondriacs confine themselves is often not less injurious to the health than the contrary indulgence.

A very ingenious chapter follows on the organs of sense, as connected in their imperfection, or the injuries they receive with the state of the mind. This concludes with a slight reference to a question, often agitated, whether the loss of sight or hearing, is the most to be regretted. The remarks here refer principally to the loss of sight.

"One case (says Dr. R.) of melancholy I well recollect, which was remarkable from the patient not having been afflicted by it until after the deprivation of his sight. Reflection upon that loss could not fail, for a time, to have been itself a source of uneasy feelings, but the continuance and gradual aggravation of his depression may be better accounted for, by his not being longer able, in consequence of this loss, to pursue his usual active employment, by its withdrawing from him the natural and exhilarating stimulus of light, and by its precluding altogether the possibility of that amusement and diversion of mind which, in general, is so constantly derived from the contemplation of external objects; to which may be added, that by confining the sensibility within a smaller compass, it condensed and increased its force. Notwithstanding all this, we find that the blind, when in society, and engaged in conversation, are in general more cheerful than other men. But, from their apparent and even actual state of spirits, when exhilarated by social intercourse, we are by no means to infer that their general condition of feeling is of the same character. Society is the proper sphere of their enjoyment. In proportion as the total obstruction of light shuts out the principal inlet to solitary amusement, they must feel delight in that which arises from a communication with their fellow-creatures. Conversation acts upon them as a dram; but when that stimulus is withdrawn, their depression is likely to be aggravated by the temporary elevation which it had induced. This, however, does not appear to be uniformly the case. I knew a man of a superior understanding, who, according to vulgar prejudice and phraseology, had the *misfortune* to be blind. The conversation happened to turn, in his presence, upon a person who was subject, without any apparent cause, to a lowness of spirits, which, though many things had been tried, nothing had been able to remove. Upon the blind man being asked what *he* thought would be most likely to cure the malady of this mental invalid, he emphatically replied, 'put out his eyes.'"

If we were called to give an opinion on these questions, we should not scruple to say, that the blind man has enjoyments which others cannot command. Rousseau, when dis-

 gusted gusted with the world, retired, and called up to his imagination an ideal set of beings as perfect as he conceived men ought to be, and as he foolishly thought himself. To do this, he was obliged to leave society, and soon found the want of it by his perpetual visits to the metropolis. The blind man, on the contrary, has all the blessings of social intercourse, and enjoys his ideal world without the necessity of retiring from society. Having nothing to distract his attention when not engaged in conversation, having his own choice in the society and in the books he wishes to hear read to him, we find him constantly with a smile on his countenance, when taking what may be called his solitary walks. If his last conversation was agreeable, he dwells on it at his leisure, without the distraction of his mind by external objects; or, if other recollections are more agreeable, devotes himself to them, or to fond anticipations, often more pleasing than the object itself when acquired.

We have dwelt so long on this interesting performance, that our limits will not permit more than a general recapitulation of the remainder.

The rest of the essays are entitled, "mental derangement not indicative of constitutional vigour of body or mind"—"physical malady the occasion of mental disorder"—a short chapter "on the atmosphere of London"—another on "dyspeptic and hepatic diseases"—a very long and interesting one on "palsy, idiotcy, and convulsions." On the next, concerning the "hereditary nature of madness," we feel constrained to regret that the peculiar force of the author's style has, in our opinion, betrayed him into something like an anathematous mode of expression, which, if not absolutely necessary, cannot be excused. We shall give his words, and then offer our objection.

"Nothing (says he) can be more obvious than that one who is aware of a decided bias in his own person towards mental derangement, ought to shun the chance of extending and of perpetuating, without any assignable limit, the ravages of so dreadful a calamity. No rites, however holy, can, under such circumstances, consecrate the conjugal union. In a case like this, marriage itself is a transgression of morality. A man who is so situated, in incurring the risk of becoming a parent, involves himself in a crime which may not improbably project its lengthened shadow, a shadow, too, which widens in proportion as it advances, over the intellect and the happiness of an indefinite succession of beings.

"The ruffian who fires at the intended object of his plunder, takes way the life of him only at whom his aim is levelled. The bullet which penetrates the heart of the unfortunate victim, does, in general, no farther mischief. But he who inflicts upon a single individual individual the worse than deadly wound of insanity, knows not the numbers to which its venom may be communicated: he poisons a public stream out of which multitudes may drink; he is the enemy, not of one man, but of mankind."

Let us reflect that no reserve of this kind is made when the *Fiat* or blessing was pronounced to our first parents, and in them to us all—"be fruitful and multiply." But we have heard it called unfair to quote Scripture in physiological arguments; and, perhaps, there may be objections to it, as it always leads to verbal controversy. We only wish then to reflect how few there are who can throw the first stone. In what family may we not trace instances of insanity. But this is only a small objection to such a theory. Whence, we would ask, originated this hereditary disposition to madness. Unless we can trace it to Noah or his children, it must commence in the offspring of a pair free from the malady; and this would at once lead to an extinction of the race, if marriage were deemed improper, lest the issue should be mad. Let us consider further, how nearly we all approach, at certain intervals, to madness, and even accuse ourselves of acting under such an impression. Still more to what but an enthusiasm bordering on madness, and, for the most part, connected with such a family propensity, do we owe most of the great and good actions which become the aggrandisement of a family, the boast of a nation, and often the melioration of the whole human race. We should not have dwelt so long on this head, had we not a practical knowledge of the moral disadvantages attending the prevalence of Dr. Reid's opinion. On the profligate and unprincipled it produces no effect. On the amiable and good, who, of all others, are the fittest to become parents, the interdiction is often viewed in a moral light, and probably the world, by that means, is deprived of individuals who might have proved its brightest ornaments.—We have thrown out these hints with a sincere wish that the author, in a "more systematic and complete" work, which we still hope to see, will consider this question more maturely, and with less regard to his early impressions.

The chapter on "old age" abounds in useful reflections. The succeeding one, on "Lunatic asylums," is a subject which has lately been so closely canvassed that we shall not add more to it. Some judicious observations follow on "counteracting the first tendency to madness," and on "lucid intervals." In the following chapter, on "bleeding," we think the author, as well as Dr. Heberden, evinces more caution than is necessary, and that the error is more frequently quently on the side different from that marked by him and his venerable predecessor; but this is mere matter of opinion.

The remaining chapters, on "pharmacy"—on "bodily exercise"—on "real as a remedy for imaginary evils"—on "occupation,"—contain useful remarks; but we cannot venture to extend our own or our extracts any further. It cannot be necessary to give any general opinion of the work, as that will be readily collected from the perusal of what we have offered.

---

*A Compendium of Medical Practice, illustrated by interesting and instructive Cases and by Practical, Pathological, and Physiological Observations.* By JAMES BEDINGFIELD, Surgeon, late Apothecary to the Bristol Infirmary. —Highley and Son. pp. 309.

THIS is a very useful book, but the title, instead of being, to speak technically, a taking title, is a real injury to it. Had it been called A Selection of Cases which occurred at the Bristol Infirmary, with the Examinations after Death, and printed on a somewhat less expensive plan, it must have found its way to most medical libraries; for we will venture to say, that few books contain more practical information, derived from the best sources. This information in the greater number of cases serves to confirm what is now pretty generally understood, namely, that in all acute diseases the practitioner is constantly to keep in view the preservation of all the organs from the dangers immediate and remote of high inflammation. Many of the pathological remarks might have been spared, but on these occasions we are never fastidious. If we were, might we not reject Morgagni himself, and still more Bonetus, who reasons gravely concerning a cancerous liver?

We shall offer a few of Mr. Bedingfield's cases as the best mode of giving the reader a correct knowledge of the whole; and, having in our last Number a very long paper on Tracheitis, we cannot do better than transcribe the examinations more immediately connected with that disease.

"*Pharyngis Ulceratio.*—Margaret Semay, aged 30 years, was admitted on the 14th of December, 1814, with the usual symptoms of pneumonia. For their relief she was bled, antimonial medicines prescribed, and a strict antiphlogistic regimen enjoined.

"Under this treatment, persisted in for fourteen days, she appeared to be nearly recovered; when she began to complain of a disagreeable sensation about the throat, attended with a disposition to cough, and a frequent discharge of frothy mucus. These symptoms gradually became more urgent and distressing; a disagreeable hoarseness succeeded, which sometimes was so great as to prevent what what she said from being understood; at other times she could only whisper faintly and indistinctly.

"Difficulty in deglutition and respiration was next experienced; deglutition occasioned much pain; the inhalation and expulsion of the air, produced a peculiar and disagreeable sound. A sound somewhat similar may be feigned by drawing air quickly and forcibly down the trachea, at the same time contracting the glottis, and making an effort to form the guttural sound ur-r-rh-r. Instead of this noise, occasionally an unpleasant whistling prevailed.

"These symptoms evidently pointed out a diminution, from some cause, in the capacity of the glottis or trachea; while the incessant cough, copious expectoration of frothy mucus, and pain felt upon handling or pressing upon the sides of the thyroid cartilage, seemed to indicate that ulceration existed upon some portion of the membrane lining the larynx.

"Upon inspection of the posterior parts of the fauces and pharynx, no trace of disease could be discovered; but the difficulty and pain experienced in swallowing, rendered it probable that some morbid change of structure existed out of sight.

"The patient remained in this state for twelve weeks, when the powers of life rapidly declined. Her pulse became almost imperceptible; respiration laborious; deglutition nearly stopped. In this state she continued four days, when she suddenly threw up a large quantity of blood from the lungs, which threatened instant suffocation. She expired within a few hours after this occurrence.

"The appearances which the parts exhibited upon dissection, afforded a satisfactory elucidation of the symptoms which existed during the life of the patient. The trachea and bronchiæ were particularly narrow.

"Just below the arytænoid cartilages, a considerable degree of inflammation had existed. Marks of increased vascularity were very apparent; a redness extended itself for the distance of an inch and an half along the posterior part of the trachea. I shall have occasion again to notice this circumstance, when the disease which existed in the pharynx is described.

"At the anterior part of the pharynx, just below the rima glottidis, a tumor in shape and size resembling a filbert, was situated. Its external surface, or that part looking towards the back of the pharynx was smooth, and it had the appearance of an absorbent gland; that portion of it (its basis) which was opposed to the anterior part of the pharynx was in a state of ulceration. By its pressure it had likewise produced an ulceration of the membrane of the pharynx and the adjacent muscles.

"This ulceration extended for a considerable way downwards, and by it an excavation had been effected, which, had the patient lived a short time longer, would have formed a communication between the pharynx, larynx, and superior part of the trachea; the membrane spread upon those parts forming the only barrier between them. This is the spot to which I alluded as being in an inflamed state. This inflammation probably induced the incessant cough with which this woman was harrassed.

"The slight partition which existed between the pharynx and larynx, and the superior parts of the trachea and œsophagus, could not be discovered, until a considerable quantity of curd-like scrofulous matter had been dislodged from the excavation.

"Several scrofulous tumours, about the size of large glandulæ Pacchionæ were met with upon the inner surface of the œsophagus. No disease was found in the lungs or abdominal viscera.

"*Laryngis Ulceratio.*—This disease is of more frequent occurrence than is generally suspected. It is often mistaken for phthisis pulmonalis; but the peculiar hoarseness and the difficulty of respiration with which it is attended, together with an almost total exemption from hectic paroxysms, are circumstances which will enable us to distinguish them from each other. Its precise nature will be best elucidated by a narration of one or two cases.

"William Birch, aged thirty-nine years, was several times admitted into the infirmary in the course of three years, labouring under symptoms of pneumonia. These symptoms were uniformly relieved by venesection, blisters upon the chest, and low diet; he complained however of a 'tickling sensation about the upper part of the throat with a constant desire to cough,' and his voice gradually underwent considerable alteration. At first it was only thick, but it became by degrees exceedingly hoarse and disagreeable.

"At the last time of his admission, the symptoms were the following. Hoarseness, a harrassing and almost incessant cough, with a very copious expectoration of a frothy mucus, in which purulent particles were plentifully interspersed; the expectorated fluid had sometimes rather the appearance of pus distended with minute air bubbles, than simple mucus.

"The pulse was frequent and rather full; the tongue generally white in its centre and florid at its edges; the uvula was seen to be elongated; the back part of the fauces very red, and the tonsils somewhat enlarged; appetite and spirits good; no other feeling of indisposition than that of lassitude, which a want of natural rest induced; for during the night the cough was so incessant as frequently to preclude sleep altogether. The body was much emaciated, the skin was sometimes dry and rather hot, and the cheeks a little flushed; but he never perspired profusely. He continued in this state four months, when the powers of life became utterly exhausted.

"A diminution existed in the superior aperture of the larynx, and its membrane was thickened and vascular; more particularly about the edges of an ulcer, by which one of the arytænoid cartilages had been completely destroyed, and a large portion of the other removed. There were no marks of disease in the trachea, and, as the ulceration within the larynx so satisfactorily accounted for the symptoms which had existed during life, the lungs were not not examined; from having however inspected several similar cases in which no disease of those organs existed, I am inclined to believe that in this case they were but little, if at all affected.

"*Laryngis et Tracheæ Ulceratio.*—Sarah Hopkins, aged nineteen years, was admitted on the 2d of June, 1814, affected with difficult respiration accompanied by a peculiar noise in the trachea somewhat resembling croup, a cough, and a soreness of the throat. Upon inspection of the fauces the uvula was observed to be elongated and the tonsils enlarged. She exhibited no marks of constitutional disease nor of debility; her pulse was perfectly natural, and she was perfectly free from pain in the chest. According to her own account, she had been in the state above described three months.

"Twelve ounces of blood were taken from the arm; a blister applied to the chest; a saline antimonial mixture taken every six hours; and a lohoc whenever the cough was troublesome.

"June 4th. The same difficulty of respiration; bowels confined. A cathartic powder was ordered to be taken immediately; and twenty drops of tincture of opium mixed with forty drops of tartarized antimonial wine at bed-time.

"Early in the morning of the 5th, she died suddenly. Her breathing had become more laborious the preceding evening.

"The rima glottidis was very much contracted and the epiglottis abraded upon its sides and concave surface.

"An ulcerated surface commencing at the superior and posterior part of the larynx extended downwards to a small distance below the ventricles of Galen.

"The trachea was filled with purulent matter. Upon this being sponged away, an extensive and deep ulceration was discovered upon its posterior part, about half an inch below the inferior aperture of the larynx. About a quarter of an inch below this, upon the anterior part of the membrane, another ulcer was situated. This ulcer spread in a circular direction and nearly embraced the whole circumference of the trachea for the space of the third of an inch. In short the whole surface of the trachea was more or less destroyed by the disease, to within about half an inch of its division into the bronchiæ. Upon this small portion there were slight traces of inflammation, and its follicular structure was very apparent.

"The membrane lining the bronchiæ was highly vascular, but no disease existed in the lungs, heart, or abdominal viscera.

"Every case of ulcerated pharynx, larynx, and trachea, which has fallen under my observation has terminated fatally, and I feel myself incapable of suggesting any perfectly satisfactory or successful mode of treatment; I suspect however that much time is lost in having recourse to constitutional remedies, instead of regarding it as a local affection.

"The disease is extremely insidious in its approaches and progress, and will often commit extensive and irremediable ravages before its existence or nature can be ascertained."

A chap-

A chapter follows on Hæmoptöe containing some pointed, and in some respects judicious, remarks on the frequent and free use of digitalis. We shall occasionally refer to this work in common with other collections. Its value stands much higher than a mere *sepulchretum* because most of the cases were long enough in the hospital to afford a thorough knowledge of the symptoms and of their correspondence with the subsequent appearances.

The following paper on a disease so frequently unmanageable does credit to the author's candour.

"*Epilepsia.*—Most sincerely do I lament my inability to furnish a successful method of treating this formidable malady, but I have known every measure which consummate ability and ingenuity could devise, rendered perfectly abortive when it has been of long duration.

"By far the greater number of epileptic cases arise from causes which we cannot ascertain, or which, if ascertained, perhaps, we do not possess the power to remedy.

"The treatment I have seen upon the whole the most efficacious was the following.

"The force of the arterial system was diminished by drawing blood from the arm in small quantities, as frequently as circumstances indicated; an active purgative was given once or twice a week, and on the intermediate days, the oxid of zinc in sufficient doses to excite nausea.

"To adults were usually prescribed five grains three or four times a day, gradually increasing the quantity to a scruple. It will seldom be found necessary to give a larger dose than this, although a larger may be taken without injury. With children it will be advisable to begin with one or two grains.

"By persisting in these measures for a length of time, in several cases of extreme obstinacy and violence, the disease was suspended; upon laying them aside it returned, and generally terminated in fatuity or death.

"I have given the spirit of turpentine in large as well as in small doses, but no decided benefit accrued from its exhibition.

"In incipient cases, where the disease depended upon the retention, suppression, or imperfect flow of the catamenial discharge, venesection employed monthly, with active purgatives and electric shocks applied to the pubic and lumbar regions, has been decidedly useful. Bleeding, instead of diminishing the interval between the paroxysms has always appeared to me to increase it.*

"When

---

"* This observation is, I believe, in opposition to generally received opinion.

"A few months ago, I bled a patient while labouring under an epileptic paroxysm with manifest advantage. In this case the heart and carotid arteries throbbed with great violence, apoplectic stertor

"When epileptic fits depend upon the presence of worms in the intestines, means must be resorted to for their speedy expulsion.

"Sometimes epileptic fits are excited by the pressure of an extraneous body upon the brain, as a spiculum of bone. If we should be fortunate enough to discover its situation, we must remove it with a trephine. A slight depression upon the surface of the cranium will sometimes lead to a detection of the injured part.

"The brains of epileptic patients will be generally found to present the same diseased appearances as are met with in persons who die of serous apoplexy."

He further remarks, that he has sometimes seen the superior part of the cerebellum with an appearance as if pus were deposited beneath the pia mater.

---

*A Memoir on the Cutting Gorget of Hawkins, (containing an Account of an Improvement in that Instrument, and Remarks on the Lateral Operation for the Stone.)* By ANTONIO SCARPA, Member of the National Institute in Italy, &c. &c. Translated by JAMES BRIGGS, Surgeon to the Public Dispensary. London. 8vo. pp. 29.

BY an advertisement prefixed, we learn that the Translator was favoured by the Author with this paper, extracted from the Transactions of the National Institute of Italy, accompanied with a request that Mr. B. would translate it. We can only express our gratitude to both.

Whoever reflects on the operative part of surgery must, we conceive, be convinced that, to a "good workman," the proper cutting instrument is the scalpel. With this, Mr. Hunter undertook to perform every operation requiring incision, and with this he operated successfully in the stone. But, as this operation must sometimes be performed by men less conscious of their anatomical knowledge, there is much propriety in every assistance that can safely be afforded them.

The professor of course adverts to the imperfections of former instruments. As the chief objection is to their complexity and consequent uncertainty, we shall only transcribe his remarks on Cheselden.

---

stertor had commenced, and I am strongly inclined to suspect, that, had not thirty ounces of blood been speedily drawn away, effusion upon the brain would have taken place. For several days after the attack the mental faculties were much impaired, and it was apprehended that the case would terminate in complete fatuity; the powers of the mind however gradually returned, and the patient is now in a fair way of recovery. January 1816.

"Cheselden,

"Cheselden, to whom alone belongs the merit of having enriched surgery with the important invention of the great lateral apparatus, in performing this operation, made use of a knife with a convex cutting edge, four lines broad, fixed upon a long handle. With this very simple instrument, he divided the prostate gland laterally through its whole length, to the depth of four or five lines; after which, by means of a slow and gradually increased dilatation of the neck of the urethra* and orifice of the bladder, he extracted large calculi without any ill consequences ensuing to his patient. It is not however so easy a matter as an inexperienced operator might perhaps imagine, to pass a knife, within the neck of the urethra, beyond the orifice of the bladder, so that in its course it may not deviate, sometimes considerably, from its lateral direction, and divide the prostate gland to a proper depth, especially at the base, which surrounds the orifice of the bladder; for the point of the knife is easily stopped in the groove of the staff, and either from the strong resistance which the firm substance of the prostate gland generally opposes to the gorget, so as to press it on the opposite side, or from the gland receding from the instrument, the surgeon is lead to suppose that he has laid this glandular body open to a sufficient depth; when in reality he has only divided the apex, and a very small part of its base.

"To render the execution of the lateral operation easier to surgeons of less experience than Cheselden, was the laudable motive which induced Hawkins to propose his gorget. He thought that two great advantages would be gained by the use of this instrument; one, for instance, of executing invariably the lateral incision of Cheselden, the other, of constantly guarding the patient through the whole course of the operation from injury of the rectum and of the *arteria pudica profunda*. Its utility as to the latter of these objects cannot be disputed, as it is evident that the convexity of the director of the instrument defends the rectum from injury, and that its cutting edge not being inclined horizontally towards the tuberosity and ramus of the ischium, but turned upwards in the direction of the longitudinal axis of the neck of the urethra, cannot wound the pudic artery. But, with respect to the first advantage, or that of executing precisely the lateral incision of Cheselden, it must be admitted, that it does not completely fulfil the intention which he proposed, not only on account of the cutting edge of his instrument not being sufficiently raised above the level of the staff to penetrate sufficiently the substance of the prostate gland, and consequently divide it to a proper depth, but because being too much turned upwards, at that part of it which is to lay open the base of the gland, it does not divide it laterally, but rather at its upper part, towards the summit of the ramus of the ischium and the arch of the pubis; an opening, of all others, in

* It may be right to remark, that Professor Scarpa objects to the term *cervix*, or neck of the bladder; and, in our opinion, with much propriety.—Edit.

the the perinæum, the most confined, and presenting the greatest impediment to the passage of the stone from the bladder. The breadth of the point of the director is, besides, so disproportionate to the diameter of the membranous part of the urethra, that from the great resistance with which it meets, the instrument may easily slip from the groove of the staff, and pass between the bladder and rectum,—a serious accident which has very often happened even in the hands of experienced surgeons."

The description of the instrument would be scarcely intelligible without the drawing, a copy of which we do not conceive ourselves authorised to give. The mode of using it will sufficiently explain the advantage the author proposes, and has himself experienced.

"The method of operating with this instrument is as follows: having introduced the staff into the bladder, the curvature of which corresponds exactly to that of the axis of the neck of the urethra and prostate gland, and the extremity of which is rather longer than that of the ordinary staff, so as to penetrate the bladder to the extent of an inch and a half, and the external incision, and opening into the membranous part of the urethra, being made in the usual manner, avoiding the bulb, the surgeon with his left hand should hold the staff firmly against the arch of the pubis, in a line perpendicular to the body of the patient; then taking hold of the gorget with his right hand, and inserting the beak in the groove of the staff, so that the convexity of the director may be directly placed over the rectum, should run the gorget on, in a line as nearly parallel as possible to the horizontal extremity of the staff situate in the bladder, not stopping until he feel that the beak of the instrument has reached the closed extremity of the groove of the staff. After having removed the staff from the bladder and urethra, and introduced the forceps upon the groove of the gorget, the latter is to be gently withdrawn upon them, in the direction in which it had been introduced. Lastly, the position of the stone being discovered, by means of the forceps, the blades are to be gently opened, and the neck of the urethra and orifice of the bladder, so far gradually dilated by them, that the operator may be able to take hold of it easily, and extract it, without bruising or lacerating the parts through which it is to pass.

"It is a certain fact, which I have ascertained by repeated observations and measurements, taken from the dead subject in the adult, that a line inclined to the axis of the neck of the urethra and prostate gland, at an angle of 69°, passes laterally through the base of the gland, at the part most convenient of all others for the extraction of the stone in the perinæum, this being neither too near the arch of the pubis, nor the inferior and posterior surface of the gland.* And, as the cutting edge of the gorget is inclined to the longitudinal

"* The prostate gland is shorter on its anterior than posterior surface; and the cervix of the urethra does not pass precisely through longitudinal axis of the director, precisely at the same angle, when the instrument is held in the direction of the natural axis of the neck of the urethra and prostate gland, it follows, from mechanical necessity, that in pressing it into the bladder in a line as nearly parallel as possible to the horizontal groove of the staff, the whole of the gland, with the orifice of the bladder, must be cut through at this precise point.*

"The staff being held firmly against the arch of the pubis, in a line perpendicular to the body of the patient, so that the convex part of the director may be placed towards the rectum, and take the exact course of the axis of the neck of the urethra and prostate gland, is an invariable guide by which the cutting edge at this determined angle must of necessity divide the gland laterally at the part most advantageous for the removal of the calculus. This rule is the more easily to be determined, and more securely observed, as the staff lodges itself, as it were, under the arch of the pubis; and as this, of all the positions which can be given to it, is the firmest and the most commodious to the surgeon, during the operation."

We have not yet heard that this instrument has been tried in London; but, that it will be, cannot be questioned, from the high reputation of the inventor and the liberality of the surgeons in the metropolis. As soon as we are acquainted with the result, we shall not fail to inform our readers.

---

### *Edinburgh Medical and Surgical Journal*, No. XLVIII. for October, 1816.

### Art. I.—*Critical Review of the State of Medicine during the last Ten Years.*

THIS paper is supposed to be the production of Prof. Kurt Sprengel, of Halle. That it is a German or Flemish production, can admit of no question.

---

through the centre of it, but through that portion of it which is nearest the arch of the pubis. On account of the greater shortness, therefore, of the cervix of the urethra, and smaller bulk of the gland, the nearest way from the membranous part of the urethra to the cavity of the bladder, would be through the anterior part of it; but as the incision made in the smaller portion of it would fall immediately under the arch of the pubis, which would present a great obstacle to the passage of the stone, the lateral incision, though carried through the longest and thickest part of it, must always be preferable to a division of it anteriorly.

"* In the construction of the instrument, therefore, great skill and accuracy are requisite on the part of the artist.

It

It commences with the history and literature of medicine. Here, as might be expected, we meet with German labours, German commentators, and German translators. Of all that is English, we have only Dr. Falconer's opinion concerning the *Morbus Cardiacus* of the ancients, published in the Memoirs of the London Medical Society, a work certainly full of learning, but, as we remarked when it first appeared, far from satisfactory in its conclusions. The most important of all the works mentioned in this division of the paper is, in our opinion, Gruner's *Itinerarium sudoris Anglici ex actis designatum.* We are surprised, considering how much our island and our countrymen in every part of the world were interested in this question, that the work was never reprinted in England, nor found its way into any of our periodical journals. This is the more remarkable, as it was written in Latin, a language well understood by the reading part of the profession.

A general view follows of all the periodical publications on medicine in Europe and America. Here it is pleasing to mark the candour of a foreigner. Whilst handsome compliments are paid to the quarterly production of Edinburgh, and the monthly journals of London, those cold-blooded trimestral and annual attempts at stifling genius which were extinguished, not for want of talent in the performers, but from the disgust with which they inspired every honest breast, are altogether unnoticed.

The anatomical division follows, in which we are pleased to find that Great Britain is treated with the same courtesy. Whilst manuals and vade-mecums are enumerated without end in Germany, those disgraceful compilations which have issued from the English press, suited only to assist the memory of the student immediately before examination, or to make him fancy he has a complete practice of medicine in his waistcoat-pocket, are left unnoticed. Some others, which are passed over, might have been mentioned; but we ought to be thankful that, whilst Sir E. Home's account of the new discovered lobe in the prostate gland, as well as his hints concerning secretion, are passed over, his lectures at the College are respectably introduced, in the division of comparative anatomy, and even in company with the justly-celebrated Cuvier.

Chemistry is almost confined to animal substances, and, with much propriety, allowed to take her place, not as the mistress, but the handmaid, of physics. Like other officious *internuncii*, it is difficult to say whether she has done more good or harm. The present, however, seems the time when she is likely, by reforming her own errors, to instruct us how

 little little assistance we can derive from her. Let not the reader suppose that we have any wish to undervalue the labours of Woolaston, Davy, Berzelius, or Bostock. To the first we owe some important analyses of diseased productions; the second is not to blame if his brilliant discoveries have led inferior talents astray; and the two latter have executed whatever they undertook with so much fidelity, as to furnish us with satisfactory data.

The microscopical observations, which, in our opinion, with much propriety, had slept since the time of Malpighi, have been, to say the best of them, innocently renewed of late. Whether the conclusions have been accurate or no, they have led to no medical advantage, nor are we by any means satisfied that "Gruithuisen's interesting observations on the globules of chyle and of pus have led to the distinction of the latter from mucus." In the healthy state of mucus there can be no danger of confounding it with pus. Under disease, no observations that we have witnessed enable us to distinguish between the secretion of a mucous membrane and of an ulcer. We ought to recollect the various forms of each, according to their state, and the progress of disease, or approach towards health: when these are duly considered, we suspect no one will question the accuracy of our London physiologists.

Nor are we at all better satisfied with the author's opinions concerning the influence of galvanism in physiology. That the torpedo and electric eel have a galvanic apparatus, no one will question. But the organs by which such effects are produced are demonstrable, and their powers of action are found to depend on life, and even to increase and diminish in proportion as health and strength, or disease and exhaustion, are apparent in other parts of the body: with life they cease. Lastly, to produce such effects at all, an apparatus is discovered, peculiar to these animals; and, in that variety of means of defence with which Nature has pleased to endow various classes of animals, is it wonderful that this should exist also? But, if we were to admit that secretion, or any combination discoverable during life, is the effect of galvanism, must we not also admit that the use and quantity of this power, and its application, must be regulated by certain organs, in order to preserve that uniformity of heat, of absorption, of oxygen, and excretion of azote, which we find under the various conditions to which the animal is exposed, and the deviations from which are so inconsiderable, compared with what we should meet with in dead or inorganic matter under circumstances in other respects similar. Two long paragraphs follow on the same same subject, which never can interest a native, still less, we should think, a student, in the country that gave birth to a Bacon, a Hervey, a Sydenham, or a J. Hunter.

"Among the physiological writers of France during this period, K. L. Dumas, P. J. Barthez, and A. Richerand, are the most important. The first, by distinguishing the physical, organic, and vital powers, and by adopting Bichât's ideas of the differences of the various systems, introduced better principles, and explained the nature of the secretions better than had been done before him. But Dumas was indebted for his arrangement, and his idea of a power of fixed position, to that excellent master of his art, Barthez, of whose work a new edition was published. A peculiar combination of chemical with dynamic principles, resting upon very multifarious hidden powers, occurs in Richerand's fourth edition.

"The Italians can boast of having produced three of the principal physiological works of this period, by Jac. Tommasini, one of the most acute and best informed authors, by Jos. Jacopi, and by Stef. Gallini.

"We have become acquainted with two British publications belonging to this department; one popular, the other idealistic."

Few of our readers are ignorant of Richerand. Bichât is not without merit, which is, however, obscured by the great pains he takes to obscure our Hunter. Had he contented himself to pursue, continue, and enlarge, the discoveries of such a predecessor, we should have learned something more than we now know, and all Europe would have been enlightened. On this we have said enough in our remarks on Bichât's work. We are not disposed to quarrel with our author for the little notice he takes of English physiologists since the days of Hunter.

Several paragraphs follow on the structure and physiology of the brain and nerves. On this subject, we wait for a fair opportunity of presenting our readers with the state of science and conjecture up to the present time. At present we shall only remark, that the Professor gives Gall the credit of reviving and improving a correct mode of examining the brain. On the subject of craneology, his remarks are, in our opinion, too general. This is, however, a mere opinion; and, for the reason above mentioned, we shall abstain, at present, from any further notice. We shall observe the same silence on the probably for ever incomprehensible subject of muscular motion. The subject of respiration is compressed with equal accuracy and judgment.

"No function (says the author) of the body was more accurately investigated in all points of view, and in all its relations, than respiration. Sömmerring and Reissein examined at the same time time the lungs, their cells, and the termination of the vessels, and taught that the par vagum, and not the intercostal nerve, supplies the bronchial vessels. For this last reason, and from experiments, M. A. Caldani concluded respiration to be voluntary. Dupuytren's experiments established the influence which the par vagum has upon the functions of the lungs, and upon the change of the colour of the blood. Even by alternate pressure upon the nerves, the red blood was darkened. Hence he concluded, that such animals die in a state of asphyxia; he also found Bichât's observations confirmed, that, after the division of the pulmonary nerve, life continues for a time. Ducrotay de Blainville extended these experiments to several classes of animals, and found, that in birds, after the division of both nerves, life not only continues for six or seven days, but that likewise the chemical properties of the blood suffer little change. These experiments were rectified by Dumas: he shewed that the arterial blood became black in consequence of the disturbance of the functions of the lungs, caused by the pain from dividing the nerves. Similar conclusions were drawn from the experiments of Provençal, and of A. G. F. Einert; from the latter of which it appeared, that the change of venous into arterial blood continues to take place even after the division, provided the air still penetrate into the lungs. By the experiments of C. Gallois, already mentioned, it was finally proved, that the impulse to the motion of the organs of respiration likewise proceed from the spinal marrow, and that in animals, after their heads were cut off, respiration could be supplied by the blowing in of air. P. H. Nysten shewed, by experiments, to what changes the chemical properties of the expired air are subject in diseases.

"Comparisons with the phenomena in other classes of animals are no where more necessary than for the explanation of respiration. Hence the observations and experiments of F. L. A. W. Sorg, and of C. L. Nitzsche, were very valuable, as explaining the difference of the changes which respiration produces in the lower classes of animals; hence likewise F. v. P. Gruithuisen, in his above-mentioned Organozoonomy, and L. Oken, in his Philosophy of Nature, justly believed direct respiration, without circulation, to consist in the gasses passing immediately into the body, but that, in the higher classes of animals, and in man in particular, the changes of the fluids in respiration are produced more by an internal activity. Hence it is necessary to limit the earlier assertion of Humphrey Davy and J. Bostock, that, even in man, oxygen, and even nitrogen, are really consumed in respiration; and that the former is not employed only for the formation of carbonic acid gas. This opinion was, indeed, totally refuted by W. Allen and W. H. Pepys, who proved, that, in man, the blood never absorbs any nitrogen in respiration, and that the whole of the oxygen is employed for the formation of carbonic acid. Humboldt, Provençal, and Configliacchi, on the contrary, pointed out the immediate passage of the gases in fishes and the lower animals, and their deposition in the swimming-bladder of the former."

Candour

Candour obliges us to make this long transcript, because some of the experiments have been repeated in London, without any acknowledgment of what was done abroad. In return, however, we are not disposed to excuse the mention, in a previous passage, of C. Gallois' experiments concerning the continuance of respiration in decapitated animals, which have been anticipated by Mr. Brodie and even by John Hunter. On the conclusion drawn by the French philosopher, we have lately had occasion to make some remarks when speaking of the productions of an English physician. Hereafter the subject will come before us in a more insulated form.

The remains of the paper, as far as we are hitherto favoured with it, are confined to the subjects of generation and nutrition. On these we shall be silent till we meet with a well-digested essay, containing the substance of what is known, and its application to pathology.—*(To be continued.)*

Art. II.—*An Experiment to ascertain the Effects produced on sound Eyes, by the application of the Discharge from Eyes affected with Ophthalmia, in its different Stages; with Remarks.* By J. MAKESY, Surgeon, 1st Battalion, 62d Regiment.

This paper does great credit to the courage, research, and candour of the author. He selected the discharge from three different patients, at different stages of the disease, and applied them to his own eye, but found no inconvenience.

"The foregoing trial (says he) is, by no means, given as a decisive proof of the *innoxious* property of ophthalmic secretions; as I am fully aware, however accurately performed, it will appear too solitary a fact to warrant any positive conclusion, or to shake the present structure of popular opinion relative to ophthalmic contagion; though, it is presumed, it may not only tend to draw nearer the bonds of analogy which exist between ophthalmia and other phlegmasiæ, but also to direct the inquiries of others to a closer examination of facts connected with the subject.

"The fact of ophthalmia making rapid progress in one battalion, while another, which appears similarly situated, is comparatively free from the disease, though much relied on, may not be a conclusive argument in favour of contagion.

"The predisposing causes of many diseases are not sufficiently obvious to come under common observation; and, in every regiment, the individuals composing which being continually subject to the same condition, and obedient to the same unvaried habits, a certain collective disposition, or general temperament, (if I may be allowed the expression,) will be gradually formed and prevail, which, which, as is observed in individuals, bestows a peculiarity of character that distinguishes corps; and, although this temperament, or distinctive feature, is, in most regiments, too subtle to be detected or described, yet it may, notwithstanding, predispose to particular diseases."

The trial is unsatisfactory to us, for a few reasons which we shall point out. First, the author informs us that, on the day on which he made the application to his own eye, a Scirocco wind prevailed. Now, the Scirocco wind, if, as has been observed in one of our former Numbers, it has all the properties of the Harmattan, might be sufficient to supersede any contagious effects, even if they existed with certainty. Next, it should be observed that the writer remarks—

"On the embarkation of the British regiments at Alexandria in Egypt, some very severe cases of ophthalmia occurred on board the transports on our passage to Messina; and I have had repeated opportunities to remark, that, on this occasion, as well as on several others on board transports, where effectual separation of those attacked was impossible, it was not the comrade, or those in the same or nearest birth, who were the next sufferers, but others in a different part of the ship, for the most part contiguous to a port-hole or open hatchway.

"On foreign service, the buildings occupied as regimental hospitals are, from necessity, frequently ill calculated to admit of the classification or separation of diseases, as perhaps, one large apartment constitutes the entire hospital. Under such circumstances, favourable for the communication of ophthalmia, if contagious, I have never seen the complaint make any progress in the other patients; and, if a solitary case of ophthalmia took place, it most generally occurred in a person whose bed was near a door, window, or some aperture which admitted a current of air, and not in the vicinity of those labouring under the disease."

These men were not exposed, however, to the causes afterwards ascribed by Mr. Makesy.

"An attentive consideration (says he) of the peculiarities which mark any situation where ophthalmia most frequently prevails, will materially tend to illustrate the causes of that complaint. To answer such an inquiry, Egypt presents to the medical observer her low plains, sandy deserts, lakes in the neighbourhood of Alexandria, and the dazzling glare reflected from the uniformly white surface which every object there presents. To these also should be added, an atmospheric temperature much above that to which Englishmen are accustomed, night dews unusually heavy, and the minute particles of hot sand which float in the air, and are raised by the lightest breeze;—causes, on the pernicious effects of which, in producing the worst cases of this disease, it is unnecessary to dwell."

 "In

"In the spring and summer months of the year 1813, ophthalmia extensively prevailed in the British garrison of Palermo. Many of the inhabitants were likewise attacked with it."

It is also well known that ophthalmia has been epidemic in regiments in England. From all which we are inclined to believe that ophthalmia is oftentimes only one form of the effect produced by crowding diseased individuals. The consequence is sometimes hospital fever in its various degrees; at others local complaints, as ulcers, ill-conditioned and spreading, cutaneous diseases, erysipelas, or ophthalmia; all which have occurred, and been remarked by different army physicians, and even under certain circumstances in civil life. What has been said is, of course, only offered by conjecture; but we conceive it ought to have been taken into the account.

The remainder of the paper is taken up with the mode of treatment, which, we doubt not, may be similar, whatever may be the primary cause, the effect depending, for the most part, on the condition and idiosyncrasy of the subject. The plan most successful was free and early evacuations.

Art. III.—*Copy of a Letter on Pharmaceutical Nomenclature, addressed to his Excellency Sir James Wylie, M.D. Physician to his Imperial Majesty Alexander.* By Robert Lyall, Doctor of Medicine and Surgery, &c.

Art. IV.—*Contributions to Diagnosis.* By Marshall Hall, M.D. &c.—*Contribution First: containing,* 1. *The Effects of the Habit of giving Opiates on the Infantine Constitution.* 2. *Inflammatory Affection of the Chest in Infants.* 3. *Inflammation of the large Intestines in Adults.* 4. *Notice on the beneficial Effect of a warm and regulated Temperature in conducting the Mercurial Course, and in the Cure of Syphilis.*

Diagnosis is, perhaps, the most important part of medicine. When a disease is known, as Sydenham observes, there is little difficulty in finding a remedy. Add to this, it is the only means, when united with prognosis, by which the public can estimate the abilities of a medical man. The habit of giving opiates to children, which, at one time, was very common in the London workhouses, is now, we believe, nearly exploded. The remarks of the author may, however, be very useful in putting us on our guard where we meet with chronic complaints in infants, attended with peculiar dullness of the faculties. The remarks on inflammation in children are equally judicious; but we cannot easily comprehend how an author, professing to teach us accuracy in diagnosis, should give us the following history:

"3. Of inflammation of the large intestines:—

"On Friday evening, Mr. W. H. aged 50, and previously subject to dyspepsia, became seized with acute pain in the hypogastric region, attended with desire and difficulty of voiding urine. He seemed to experience relief by taking a little sp. æth. nitros. The next morning his complaints were renewed, and they increased through the day. In the evening there was violent pain of the hypogastric region, inducing writhing of the body, and still attended with urgent desire to void urine, with ineffectual efforts. This violent attack of pain seemed to have been immediately induced by the operation of a dose of oleum ricini. The pulse and tongue remained natural. Urine was voided, and ease obtained, on coming out of the warm bath.

"On the Sunday, the pain was felt extending generally over the abdomen. The desire to void urine continued, but the bladder was found empty on passing the catheter. No further alvine evacuation. Pulse nearly natural.

"On Monday, there had been copious evacuations by stool, and some high-coloured urine had been passed. The pulse was 90, soft and regular; the tongue white.

"On Tuesday, the pain seemed to have been again induced by the scanty operation of a saline opening medicine. A particular pain was now distinctly referred to a spot in the left iliac region, increased by pressure, and attended with a more general pain of the abdomen. No writhing of the body, but a degree of restlessness, manifested by throwing about the hands and arms; much flatus on the stomach; a little vomiting, for the first time, on taking any thing; no continued nausea or retching. Pulse 96, in the evening 84, soft and regular; the pain of the abdomen continuing; tongue white and loaded.

"About four A. M. on Wednesday, the 6th day of the disease, the hands were observed to be livid and cold, and the body to be covered with a profuse cold sweat. At seven A. M. the pulse was 124 and small. A fixed pain, similar to that formerly described as felt in the left iliac region, was now distinctly described, as affecting this region on the right side, the former pain having now nearly ceased. The patient was affected with a fallen countenance, a general coldness, and profuse perspiration, clamminess of the mouth, and change of voice. The pulse became gradually smaller and indistinct, and the patient expired at four P. M.

"On examination of the abdomen, there appeared much exudation and tender adhesions over the surface of the bowels. The ileum, cæcum, and colon, were injected with numerous blood-vessels; in some parts so as to acquire a dark colour, but the texture remained entire and firm. The appendiculæ pinguedinosæ were injected and covered with a viscid effusion, communicating the appearance of a mass of disease. The external and posterior portion of of the bladder appeared also a little injected; its internal surface was perfectly natural. The stomach, duodenum, jejunum; the liver and spleen; the heart and lungs, appeared natural and uninflamed.

"This case, which the author has endeavoured to describe with the utmost accuracy and succinctness, seems to have been a spasmodic affection, succeeded by inflammation of the large intestines, and perhaps, in a slight degree, of the bladder. Its principal features were the following: At first, violent pain in the hypogastric region and lower part of the abdomen, inducing writhing of the body, and attended with strangury and constant desire to void urine. The operation of medicine was effectual, but induced the most violent pain. No vomiting. Pulse natural. Afterwards, there was violent fixed pain, referred to a particular spot, tender under pressure, without writhing of the body, but with an appearance of restlessness. Less strangury. Tenesmus; the ready but painful operation of medicine on the bowels. Little or no vomiting. Pulse little affected until the appearance of cold sweat. A peculiar expression of countenance, induced by the action of the depressores angulorum oris.

"Dr. Willan, in the Diseases of London, has some excellent observations on the diagnosis of the affection in question. He observes, that 'mistakes arise respecting inflammation of the lower intestines, or of the colon about its connections with the cæcum or rectum. It does not, as in the case of inflammation of the ileum, or of any part of the smaller intestines, occasion, by excruciating pains, instant debility and depression, with vomiting, cold sweats, &c. There is at first a local but moderate pain, somewhat aggravated by pressure, and attended with thirst and general uneasiness. This pain seems afterwards to diffuse itself, producing strong contractions of the bowels and abdominal muscles, which recur from time to time, but have considerable intervals of ease and tranquillity. The disorder differs, however, from the colic, in this respect, that it is not attended with obstinate costiveness, and that after sufficient evacuations the pain is not mitigated. On the other hand, as the intestine is tender, and probably contracted about the seat of inflammation, a most severe pain is often excited by the operation of the mildest purgative. The pulse may at the beginning be hard and contracted, but it soon becomes weak, small, and perhaps irregular. There is a fur upon the tongue, somewhat thick, and of a whitish colour. The urine has a smooth pink sediment, which, as the disorder advances, changes its colour, and resembles a rough cretaceous powder. Vomiting is not a constant symptom in this form of enteritis."

With these judicious remarks of Dr. Willan's before him, why should Dr. Hall speak of a *spasmodic affection*, succeeded by inflammation, &c.? Was not the first symptom "acute pain with a desire and difficulty of making water?" Could diagnosis afford stronger proofs of acute inflammation in the

 pelvic pelvic viscera?—We shall not dwell on this subject, but conclude with an expression of doubt whether typhus or spasm has been the most mischievous word in the language of medicine.

The beneficial effects of confinement and regulated temperature during the use of mercury in the cure of syphilis, have been too long admitted to require any particular notice.

Art. V.—*Case of Re-union of a separated Portion of the Finger;* by Mr. James Braid, Surgeon at Leadhills.

These and other cases which have lately occurred, show that on all occasions attempts should at least be made to re-unite divided surfaces.

Art. VI.—*A Case, disproving the Doctrine, that the Surfaces of a Wound in a State of Suppuration will not re-unite by the first Intention.* By William Balfour, M.D. Edinburgh.

It is absolutely necessary that men should understand one another before they attempt to disprove. In the southern metropolis, union by the first intent is the inosculation of divided vessels; and that such really takes place, we have demonstrative proof, after the division of a vessel in the blood-shot eye, which, on the following day, we often find re-united. But, when suppuration has taken place, the action and configuration of parts is altered. In these cases, however, union may take place in two ways; by coagulated lymph if the parts are clean and excited to inflammation, or by granulations if they comein to contact, each on the suppurating surface. The present case seems a compound of both, but certainly not a case of union by the first intention.

Art. VII.—*Observations, with Cases, illustrative of a New, Simple, and Expeditious Mode of curing Gout;* by Wm. Balfour, M.D. Edinburgh.

We shall give one of these cases without any remarks of the author or of our own.

"Case I.—On the 2d of July, I was requested to visit Mr. N. M. aged 40, and of a full habit of body, whom I found labouring under a severe fit of gout. About ten or twelve days before I saw him, he had taken a very quick ride of about ten miles, by which he was greatly overheated, and took not the least care of himself afterwards. About two o'clock next morning, he awoke with violent pain in the balls of both great toes, reaching upwards along the upper part of the foot to the ankles; and, in the back part of the leg, to where the gastrocnemius muscle terminates in its tendon.

"When

"When I first saw him, the balls of the toes were still much swelled, pained, red, tense, and shining; and motion of the joints impracticable. The whole upper part of the foot, particularly about the roots of the toes and outer ankles, was œdematous. The legs were generally swelled as high as the calf; and along the tendo Achillis, especially at its commencement, very painful. The patient was very lame. He described himself as having had a paroxysm every night from the commencement of the complaint,—as the pain and heat were, in the night time, intolerable. Had taken no medicine; pulse 80; no appetite.

"I applied compression to the balls of the toes; friction to the œdematous parts; percussion to the ankles; and friction and percussion to the legs,—surrounding all the parts, afterwards, with a roller. The patient walked better immediately. Ordered a brisk purgative of decoction of senna and Epsom salts.

"3d.—Medicine operated smartly. Underwent the same treatment this morning as yesterday, with increased advantage.

"4th.—All the symptoms declining; and walking greatly improved.

"Put on his usual shoe on one foot this morning, after the operation, without being desired.—Is altogether so much better as not to need my attendance any longer.—I notwithstanding made two or three more calls, to ascertain the permanency of the cure.

"This is the first case of pure regular gout, in which I applied compression and percussion; and it is the first instance, so far as I know, of any attempt to alleviate, or to cure gout by mechanical means. My almost instantaneous, and complete, and permanent success, surprised me; but it surprised my patient more. He had suffered so long before I was called, from a conviction that no earthly power could be of any avail in gout; and it was in compliance only with the solicitations and entreaties of his friends, that he submitted to call medical aid at all. By the time, however, that I had made him the third visit, he was of quite a different mind."

### Art. VIII.—*Cases of Laryngitis*; by Ninian Hill, M.D. Greenock.

The first of these interesting and well related cases was in a female of an athletic but spare habit, who had caught cold by attending her children under scarlatina. The disease was of course suspected to have arisen from that contagion. But nothing like slough or ulceration appeared, either during life or in the examination *post mortem*. The patient lived to the fourth day, on the morning of which,

"Appearances in the fauces much the same; hawking and spitting of mucus continue. Skin hot; tongue foul; pulse strong and hard; catharsis continues; blood sizy and firm. She was again bled to the extent of about ten or twelve ounces. At three o'clock in the afternoon, she expressed herself as being greatly better, having having been able to swallow more easily, and her breathing being more free, though still noisy. But, on looking at her face, I was much struck with the sudden change. Her features were shrunk; her countenance ghastly pale; her skin and extremities cold, and bedewed with a clammy sweat; her pulse feeble and tremulous, and so quick as not to be measured. Stimulants, cordials, and warm applications, were assiduously administered, in the hope of bringing about re-action, but in vain,—she expired about six o'clock.

"The body was examined eighteen hours after death. The whole abdominal viscera were sound. Upon removing the sternum, the lungs remained distended, but there was not the least mark of disease, not even the slightest adhesion to the pleura costalis. The heart and pericardium were natural. On slitting up the trachea, there was no appearance of inflammation, or effusion. Our attention was next directed to the gullet, in hope of finding the *causa morbi*. To get at this, the trachea was laid hold of, and pulled downwards; the larynx was then cut across, close by the superior cornua of the thyroid cartilage, the divided portions of which were found to be so thickened, and so distended with healthy pus, that there was but a small aperture left for carrying on the function of respiration.

"On making this discovery, we were prevented from prosecuting our dissection farther, by the relations who were present."

This case proves, that the death of the patient was not from suffocation, and that suppuration is not always sufficient effectually to arrest even the rapid progress to dissolution.

The second case is remarkable from the disease appearing to have been excited by external violence.

"J. H. skipper, æt. 40, complains of sore throat, which I was called to examine late in the afternoon of Saturday the 6th of January, and found it red and inflamed, without any perceptible swelling of the internal or external fauces to account for the difficulty of deglutition and pain which he seemed to suffer. The febrile symptoms were such as are met with in a common inflamed throat. Pulse 112, soft and compressible; skin rather hot; tongue foul, but moist; thirst moderate; bowels natural. The following history of his complaint was corroborated by his wife. On Thursday evening, while eating a bit of fish, one of the small bones stuck in his throat, which he endeavoured to displace by hawking and coughing forcibly; swallowing at times bread imperfectly manducated, &c. Next day he attended to the discharging of his vessel; spoke, ate, and drank as usual, yet was occasionally annoyed by the bone pricking him. About four in the afternoon, anxious to get rid of a continued annoyance, he thrust his forefinger two or three times down his throat. In one of these attempts he brought on a violent fit of coughing, which forced up the bone, and he threw it into the fire; but says, that it was about an inch in length, having a forked extremity, which was coloured to the extent extent of a line or two with blood. On Saturday morning he began to feel the usual symptoms of cynanche tonsillaris, and thought he would get soon clear of it by keeping in bed; and I, though requested by his wife to see him without his consent, having no suspicion from the symptoms I witnessed of any other affection, directed her to treat it as such. However, when about to leave the room, I was fortunate in hearing the patient emit an uncommon sound in the act of inspiration, and of this I was only sensible when he inspired more fully than common. On being asked if he had any difficulty in breathing?—he said, 'a little.' In regard to the sound of his voice nothing was remarked. These last mentioned symptoms alarmed, and informed me that I had something more formidable than a common cynanche to deal with. In this view of the case ℥xviii. or xx. of blood were taken in a full stream from the arm; a saline purge; a blister for the throat; and the usual auxiliaries of gargling and inhaling were directed; and I intended seeing him early in the evening. An hour had not elapsed when I was requested to see him, as he was like to be suffocated for want of breath; and the attendants were so alarmed, that I was met on my way by several of them, urging me to make haste, as they thought him dying.

"He breathed so high that I heard him before I got to his bedroom, where I found him sitting on his bed-side, unable to speak, supporting his body, which was inclined forward, with his hands upon his knees, making strong efforts to inflate the lungs. The air passing down the larynx caused a harsh sound, like to the hoop in children ill of pertussis, only it quivered. The expirations were free from sound. His eyes were staring; face pale; and he seemed much under the influence of fear. The pulse was new, hard, and quick. The orifice was again opened and enlarged, and nearly ℔ij. of blood were abstracted before he got faint and resumed the horizontal posture. The violent motion of the respiratory muscles ceased, his breathing became easy and free from sound altogether, so long as he continued faint; in a few minutes his skin relaxed, and a copious perspiration was diffused over his body.

"Seeing him wonderfully relieved, we embraced the opportunity of urging him to swallow the salt, for the pain and difficulty of deglutition were so formidable to him, that he frequently took the solution to his mouth, and put it back again; however, with some persuasion and an effort, he got over the greater part of it; but, from the repeated painful exertions it would cost him before he swallowed the whole of it, a powder of jalap and calomel, mixed in jelly, was, with some care, swallowed at a single effort. The patient being desired to point to the part of the throat that was most painful, placed a finger on each side of the thyroid cartilage, and moved them over a space of half an inch; when this was pressed it did not seem to be more sensible than usual. Carrying the fingers to the parts immediately behind the angles of the inferior maxilla, gave most acute pain; yet there was no swelling or enlargement of the glands to be felt, or any apparent fulness visible.

"In

"In looking at the internal fauces, and bringing the root of the tongue forward with the handle of a spoon, retching was excited, which elevated the larynx, and presented the epiglottis erect and swollen, having the appearance of a ripe cherry of the most brilliant red. This and every other examination caused a fit of dyspnœa and threatening suffocation, which lasted for some minutes. His voice could not be raised above a whisper. Fifteen leeches were placed on the anterior part of the trachea, previous to the application of the blister. At six in the evening breathing sonorous, yet he seems to inflate the lungs without much effort. Pulse 104; skin moist; leech bites oozing freely. Had an enema to hasten the action of the cathartic. Ten at night, has had no difficult fit of breathing, unless he attempts to swallow, and it goes off in a few minutes. Deglutition completely obstructed."

From this time, though some few severe paroxysms occurred, threatening strangulation, yet they were of short continuance. The topical bleeding was repeated, and laudanum given freely, and the patient gradually recovered. During the complaint, one or two spots of coagulated lymph, about the size of an aphthous crust, was discovered on the glottis.

The third subject was a plethoric female, twenty-four years of age. She lost ℥xvi. of blood from the arm on the first day, xij. on the second, besides the application of eight leeches. All the particulars of treatment are enumerated in the author's final remarks; in which he discusses the suggestion we ventured to broach, that tracheitis and croup are the same disease, only differing in their phenomena from the difference of age.

"It is true (says he) that the membrane in the trachea peculiar to croup has not been found in the adult; but, as has been suggested by the ingenious editor of the London Medical Journal, in the number for May last, this may be owing to the difference of age; the effusion of lymph or adhesive inflammation being more common in the earlier periods of life. And here I may mention, that, in two dissections I lately had of children, from three to four years old, who died of croup, I found the membrane lying loose on the back part of, and extending a little way down, the trachea. It was extremely soft, and could hardly be discerned to have been tubular, and occupied so small a space as by no means to be considered as the cause of suffocation; but when the bronchi were examined they were completely filled with a viscid purulent fluid. But, whatever difference of opinion may prevail as to the nature or diagnostic of the disease, notwithstanding the few cases we have on record, the majority is in favour of the practice that has been adopted in these cases I have submitted. Early depletion, both general and local, proportioned to the urgency of the symptoms, the age and strength of the patient, must be our principal dependence. This is fully evinced from the particulars in Case I., though the the time was most unfortunately lost, owing to the particular circumstances under which the patient was placed; circumstances that would have, perhaps, misled a more experienced practitioner.

"In Case II. an opiate was given with happy effect, but not until evacuations had been pushed to the most justifiable extent.

"In Case III. large doses of calomel were prescribed, but little advantage, I apprehend, accrued from them; the patient was peevish and disobedient; she submitted to almost every thing that was done for her, with reluctance; several of the doses were afterwards found concealed about her bed, and there were no apparent effects from the mercury, so that, like J. H., she owes her recovery entirely to depletion."

Art. IX.—*Notes on the Swelling of the Tops of the Hands and Feet, described by Dr. Underwood as a Symptom of painful Dentition; and on a Spasmodic Affection of the Thumbs and Toes, which very commonly attends it.* By George Kelly, M.D. Leith.

This is a useful paper in keeping the practitioner in mind of a sympathy from dentition, which, as it is more rare than many others, is too often overlooked, or ascribed to other causes.

Art. X.—*Severe Case of Chorea Sancti Viti;* by David Lithgow, M.D. Coleraine, Ireland.

This case yielded to frequent strong purges.

Art. XI.—*An Apology for a Fact, and an Hypothesis; with Remarks on some few Passages in "Mr. Laurence's Introduction to Comparative Anatomy and Physiology."* By James Woodham, Surgeon of London.

This paper may be called a *review* of Mr. Laurence's Lectures. It has a considerable share of merit, and probably we may, in a future *Collectanea*, transcribe it, with some extracts from the Lecture. In doing this, we may fulfil the office of a Reviewer without trouble or responsibility; both of them important considerations in subjects bordering upon metaphysics.

